# A multigene model for response stratification to neoadjuvant chemotherapy in triple negative breast cancer

**DOI:** 10.1016/j.breast.2026.104764

**Published:** 2026-03-17

**Authors:** Nadine S. van den Ende, Marcel Smid, John W.M. Martens, Reno Debets, Agnes Jager, Carolien H.M. van Deurzen

**Affiliations:** a Department of Pathology, Erasmus MC Cancer Institute, Erasmus University Medical Centre, Rotterdam, the Netherlands; b Department of Medical Oncology, Erasmus MC Cancer Institute, Erasmus University Medical Centre, Rotterdam, the Netherlands

**Keywords:** Triple negative breast cancer, TILs, HER2-Low, Angioinvasion, Gene-expression, Gene classifier

## Abstract

Around half of triple negative breast cancer (TNBC) patients achieve a pathological complete response (pCR) based on neoadjuvant chemotherapy (NAC), which is associated with a good outcome. Conversely, in patients with a poor response to NAC, there is a clear need to administer more effective therapeutic strategies. Accurate prediction of tumor response could enable the implementation of more personalized and effective treatment strategies.

In this retrospective multicenter study, formalin-fixed paraffin-embedded tissues of pre-NAC needle biopsies from TNBC patients treated between 2013 and 2022 were analyzed. Clinical, pathological, and transcriptomic data were combined in a prediction model, using a leave-one-out design, to predict the response to NAC, followed by external validation in an independent dataset.

In total, 204 patients were included, comprising 87 good responders and 117 poor responders. A transcriptomic based prediction model showed that all samples but one clustered correctly in the good or the poor responder category. External validation showed an accuracy of 85% in predicting a good response to NAC, using a 31-gene signature. On the other hand, prediction of having a non-pCR was not substantial in this external cohort, since only 58% were predicted correctly.

This study suggests that a 31-gene prediction model may help identify TNBC patients who are likely to achieve a pCR following NAC alone. These patients may not require therapeutic intensification, such as addition of immunotherapy, thereby minimizing exposure to unnecessary treatment-related toxicity and reducing associated healthcare costs. Nonetheless, further optimization and prospective validation are needed prior to moving towards clinical implementation.

## Highlights

• 204 TNBC biopsies studies, comparing pCR and very poor response (RCB3-like).
• A transcriptomic model clustered nearly all patients as good or poor responders.
• External validation: 85% accuracy in good, 58% in poor, due to low RCB3-like share.
• Model may help identify pCR patients who can avoid extra therapy.

## 1 Introduction

Triple-negative breast cancer (TNBC) is a clinically aggressive breast cancer (BC) subtype, characterized by the lack of the estrogen receptor (ER), progesterone receptor (PR), and human epidermal growth factor receptor 2 (HER2) expression [1,2]. TNBC is associated with a higher risk of early recurrence and metastases compared to other BC subtypes, emphasizing the need for more durable and targeted therapeutic strategies [[3], [4], [5]].

The absence of the three actionable receptors renders patients with TNBC ineligible to endocrine or HER2-targeting therapy. Therefore, chemotherapy remains the standard of care treatment, which is often given in the neoadjuvant setting [6,7]. Currently, it is estimated that around 50% of TNBC patients achieve a pathological complete response (pCR) after neoadjuvant chemotherapy (NAC), which has emerged as a surrogate marker for long term outcomes [6,8]. On the other hand, there is a subset of TNBC patients that show chemoresistance to the standard of care treatment, resulting in relatively high recurrence rates and increased risk of metastasis.

The pivotal KEYNOTE-522 trial demonstrated that adding pembrolizumab to platinum-taxane-based NAC significantly increased pCR rates from 56% to 63% and improved event-free survival (EFS) and overall survival (5-year EFS: 92.2% versus 88.2%; HR 0.63) [6]. However, although these data show that a subset of patients derive benefit from adding pembrolizumab, most of TNBC patients do not gain additional advantage beyond that achieved with chemotherapy alone. Retrospective biomarker studies suggest that tumor-infiltrating lymphocytes (TILs) and tumor mutational burden may predict response to NAC, although standardization across cohorts is lacking, underscoring the need for more precise identification of patients who are likely to respond well to NAC [4,6].

In hormone receptor positive/HER2 negative BC, genomic assays such as Oncotype DX and MammaPrint are routinely used to refine recurrence risk and guide chemotherapy decisions [9]. For HER2 positive BC, gene expression-based assays are currently in development, like HER2DX, which can help to inform prognosis and treatment escalation or de-escalation strategies [10]. A similar approach is needed in TNBC, to guide treatment intensity.

Among TNBC patients demonstrating a favorable response to standard NAC alone, therapeutic de-escalation strategies, such as omitting immunotherapy, may be justified to avoid unnecessary, often irreversible, immune-related toxicity and associated healthcare expenditures. Conversely, in patients with a poor response to standard NAC, there is a clear need to administer more effective therapeutic strategies. Therefore, accurate prediction of tumor response based on the pre-NAC biopsy would enable the implementation of more personalized treatment strategies. In this national multicenter study, we aimed to generate a treatment response prediction model for TNBC patients based on the tumor phenotype of the pre-treatment biopsy.

## 2 Methods

### 2.1 Patient and tumor characteristics

TNBC patients were selected via the Dutch Nationwide Pathology Databank (Palga) and included based on predefined inclusion criteria and tissue availability [11]. Formalin-fixed paraffin-embedded (FFPE) tissue blocks of the pre-treatment needle biopsies were collected of good and poor responders to standard NAC. Patients were considered good responders when a pCR was confirmed after NAC, no locoregional or distant metastasis were present during follow-up until inclusion (December 2023), and the patients were still alive at the date of inclusion. A pCR was defined as no invasive residual tumor cells in the breast and axillary lymph nodes, corresponding to RCB-0 (ypT0/is ypN0). Poor responders were defined as patients with 50% or more residual tumor after NAC (corresponding to RCB-2 or RCB-3 disease) and a pathologically proven distant metastasis during follow-up. Clinical information was requested via the Dutch Cancer Registry. This resulted in a retrospective national cohort of primary TNBC patients, diagnosed between 2013 and 2022 in 30 centers. Coded, leftover patient material was used in accordance with the Code of Conduct of the Federation of Medical Scientific Societies in the Netherlands [12]. According to these national guidelines, this work was not subject to the Medical Research Involving Human Subjects Act (WMO; METC 02.593).

Central pathology review of whole tissue slides included histologic subtype, grade, angioinvasion, Ki-67 expression, and density of TILs, according to the recommendations of the International TILs Working Group [13]. ER, PR, and HER2 status were based on the original pathology report. Tumors were considered TNBC when ER and PR levels were below the 10% cutoff, according to Dutch guidelines [12]. Since the international ASCO/CAP guidelines follow a 1% cutoff, the numbers of ER-0 (0%) versus ER-low (1-9%) were also provided [14]. HER2 status was determined according to international ASCO/CAP and ESMO guidelines [15,16]. Ki-67 expression was based on the percentage of positive tumor cells within the whole tumor area, according to the International Ki-67 in Breast Cancer Working Group [17].

### 2.2 RNA isolation, sequencing and data processing

RNA isolation of micro dissected tumor and tumor associated microenvironment was performed following standardized and previously described protocols [18,19]. RNA sequencing was performed on the Illumina Novoseq6000 platform at Novogene (Cambridge, United Kingdom), using the FFPE sample Eukaryotic RNA-seq library preparation (250-300 bp insert strand specific library with rRNA removal) and generating 150 bp paired-end reads. Raw fastq files were trimmed and aligned to the human reference genome (GRCh38) using STAR (v2.7.9). Duplicates were marked and resulting bam files indexed using Sambamba (v0.8.1). GENCODE Release 45 (https://www.gencodegenes.org/) was used for gene annotation. The R package Rsubread (v2.16.1) was used to obtain raw read counts, which were normalized using GeTMM [20]. This led to sequencing data that was captured for 78 samples (40 good and 38 poor responders) with sufficient quality i.e. more than 21,000 mapped genes and a duplication rate <46%. No bias was shown via principal component analysis for the RNA expression distribution in age of the biopsy, originating hospital or poor/good response status. Gene expression levels were normalized and used for subsequent analysis.

### 2.3 Prediction model analysis based on transcriptomic data

To investigate if a sufficiently powered gene expression signature was present in our cohort, an initial leave-one-out (LOO) design was used. In each iteration, one sample is left out with the remaining samples used as a test set in a penalized multivariate regression model (least absolute shrinkage and selection operator, LASSO) using the GRidge R package (v1.7.5). A 10-fold cross-validation within the test samples provides an optimal shrinkage penalty. Next, the selected genes from the test set are used to calculate the probability that the left-out sample is a poor prognosis case. This prediction (probability <50% indicates good prognosis) is then compared with the true status of the sample. All samples will receive a probability/prediction, and the overall sensitivity and specificity to predict good prognosis is established. The genes selected in each LOO iteration were exported to determine how often each gene was included in the signature. In addition to the LOO prediction model, an RNA-histopathology (H&E and immunohistochemistry (IHC)) combination model was conducted. This model included the multivariate IHC characteristics and a selection of 25 genes from the LOO procedure; those that were used in at least 50 test sets. These 25 genes were combined in a single ‘delta’ score; the difference between the average expression of the genes that were upregulated in the poor responders minus the average expression of downregulated genes in poor responders.

Encouraged by the results in our cohort, an independent external validation was performed by using our RNA sequencing subset as training dataset (n = 78) and a publicly available dataset reported by Loibl et al. as test set (n = 482) [21]. The cohorts were processed differently, which required additional steps to ensure expression values were comparable. First, data were matched to only use genes that were available within both cohorts. Next, within each separate cohort data were standardized using a z-score normalization per gene. Using again a LASSO model, a 31-gene signature was generated from the training set. This signature was validated once in the test set; the LASSO beta values were used to calculate the probability a test sample was a good responder. A predicted probability >0.7 was used to categorize the test samples as ‘responders’, indicating the good responders with a pCR. Below the set cut-off of 0.7 the test samples were predicted as non-responders with residual disease (Residual Cancer Burden score 1, 2 or 3).

### 2.4 Statistical analysis

Statistical analyses were performed using IBM SPPS Statistics version 26 and R (version 4.3.2). The Pearson Chi-square or Fisher's exact tests were used to investigate differences between the good and poor responders to NAC for the categorical variables. A Mann-Whitney *U* test was performed for continuous variables without a normal distribution. Multivariate logistic regression analysis was performed to analyze which of the univariate significant variables were independently associated with response to NAC. A two-sided p-value below 0.05 was considered statistically significant.

## 3 Results

### 3.1 Independent morphologic and IHC predictors for the response to NAC

**Fig. 1:**
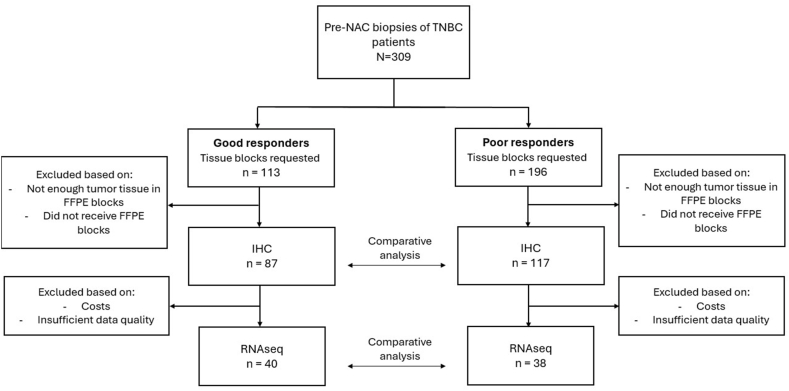
Flowchart of the inclusion and exclusion of biopsies in this study. NAC = neoadjuvant chemotherapy; TNBC = triple negative breast cancer; IHC = immunohistochemistry; RNAseq = RNA sequenced.

Patient- and tumor characteristics of the 204 cases were compared between good (n = 87) and poor responders (n = 117) (Fig. 1 and Table 1). The good responders had more often a smaller tumor and were more often node negative compared to the poor responders (p < 0.001 for both variables). The histologic subtype differed significantly, where the no special subtype was more often diagnosed in the good responders, while the rate of lobular and metaplastic carcinoma was higher in the poor responders (p = 0.015). The Ki-67 expression was significantly higher in the good versus the poor responder group (median 65% versus 55%; p = 0.001). This indicates that good responders have higher proliferation activity, which is supported by a non-significant trend towards higher grade and mitotic count. Moreover, less angioinvasion and a higher rate of a HER2-0 score was observed in the good compared to the poor responders (p = 0.003 and p < 0.001 respectively). The median density of TILs was significantly higher in good responders compared to poor responders (12% versus 7%; p = 0.005). Multivariate logistic regression showed that clinical node negative status, absence of angioinvasion, HER2-0 status and a higher median density of TILs were independently associated with a good response to NAC (p = 0.026, p = 0.047, p < 0.001, and p = 0.009 respectively).

**Table 1:** Clinicopathologic characteristics based on the pre-NAC needle biopsy.

Characteristics	Good responders (n = 87, 42%)	Poor responders (n = 117, 58%)	P-value univariate	P-value multivariate
Age				
Median in yr (range)	50 (31-72)	49 (24-81)	0.260^a^	
Clinical T stage				
T1	23 (26%)	7 (6%)	**<0.001**^b^	0.243
T2	51 (59%)	53 (45%)		
T3	7 (8%)	31 (26.5%)		
T4	0 (0%)	17 (14.5%)		
Unknown	6 (7%)	9 (8%)		
Clinical N stage				
Node negative	72 (83%)	32 (27%)	**<0.001**^c^	**0.026**
Node positive	8 (9%)	72 (62%)		
Unknown	7 (8%)	13 (11%)		
Clinical stage				
I (IA – IB)	20 (23%)	3 (3%)	**<0.001**^b^	0.117
II (IIA – IIB)	59 (68%)	35 (30%)		
III (IIIA – IIIC)	2 (2%)	52 (44%)		
	0 (0%)	18 (15%)		
Unknown	6 (7%)	9 (8%)		
Histologic subtype				
No Special Type	84 (97%)	101 (86%)	**0.015**^b^	0.877
Lobular carcinoma	0 (0%)	9 (8%)		
Metaplastic carcinoma	1 (1%)	4 (3%)		
Other	2 (2%)	1 (1%)		
Unknown	0 (0%)	2 (2%)		
Histologic grade				
Grade 1	0 (0%)	1 (1%)	0.058^b^	
Grade 2	8 (9%)	22 (19%)		
Grade 3	78 (90%)	90 (77%)		
Unknown	1 (1%)	4 (3%)		
Mitotic count				
Median (range)	17 (4-38)	15 (1-50)	0.196^a^	
Angioinvasion				
Not present	86 (99%)	102 (87%)	**0.003**^c^	**0.047**
Present	1 (1%)	13 (11%)		
Unknown	0 (0%)	2 (2%)		
Ki-67 expression (IHC)				
Median percentage (range)	65 (12-95)	55 (3-90)	**0.001**^a^	0.287
Low (<20%)	3 (3%)	10 (9%)	**0.013**^b^	
Intermediate (20-60%)	33 (38%)	60 (51%)		
High (>60%)	45 (52%)	38 (32%)		
Unknown	6 (7%)	9 (8%)		
Estrogen receptor				
Negative (0%)	44 (51%)	76 (65%)	0.391^c^	
Low (1-9%)	13 (15%)	15 (13%)		
Unknown percentage	30 (34%)	26 (22%)		
Progesteron receptor				
Negative (0%)	49 (56%)	80 (68%)	0.803^c^	
Low (1-9%)	8 (10%)	11 (10%)		
Unknown percentage	30 (34%)	26 (22%)		
HER2 status				
HER2-0	83 (95.5%)	47 (40%)	**<0.001**^c^	**<0.001**
HER2-low	3 (3.5%)	52 (45%)		
Unknown score	1 (1%)	18 (15%)		
Stromal TILs				
Median percentage (range)	12 (1-80)	7 (1-80)	**0.005**^a^	**0.009**

Unknown values are excluded from p-value analysis.

a Mann-Whitney *U* test.

b Chi-square test.

c Fisher's exact test.

### 3.2 Treatment regimen

Detailed treatment data was known for 93% and 94% of good and poor responders, respectively. All good responders with known treatment data received both anthracycline and taxane therapy, whereas 89 poor responders (81%) received both treatments (supplementary file 1). Only three poor responder patients received neoadjuvant immunotherapy. In the overall cohort, good responders were treated more frequently with cyclophosphamide, anthracyclines, taxanes and platinum agents compared to the poor responders (p < 0.001, p = 0.003, p < 0.001, and p = 0.002, respectively). After multivariate analysis, only the addition of cyclophosphamide was significantly associated with a good response to NAC (p < 0.001).

In line with this, within the RNA sequencing subset, significantly more good responders were treated with cyclophosphamide and platinum agents compared to the poor responders (p < 0.001, and p = 0.01 respectively).

### 3.3 Class prediction of response to NAC

**Fig. 2:**
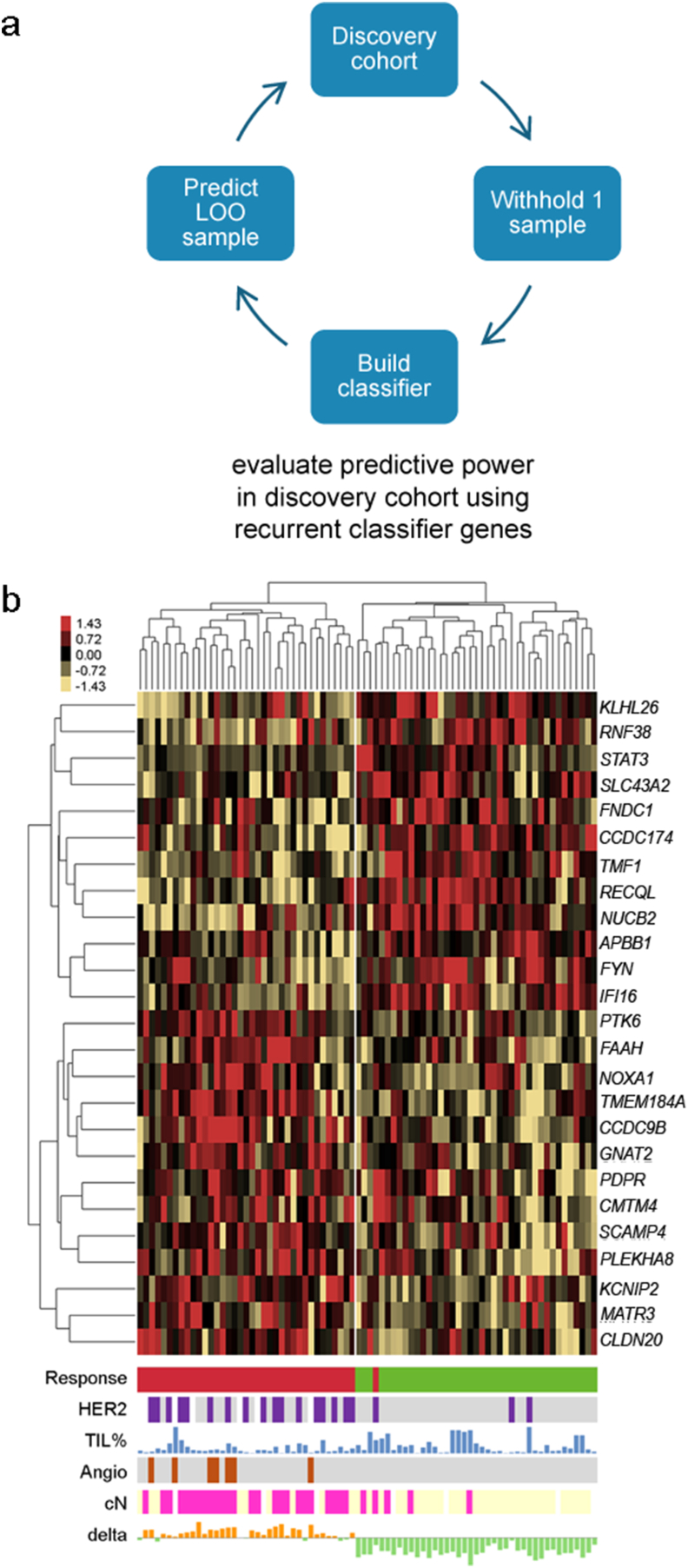
A 25-gene prediction model based on our dataset. A) Schematic overview of the LOO design to develop the prediction model. B) Heatmap after hierarchical clustering of 25 genes selected from the prediction model. Expression was standardized with red/yellow in the heatmap indicating z-values above/below 0, respectively (and thus higher/lower expression). The first track below the heatmap indicates poor/good response (red = poor/green = good). Track 2, 3, 4, and 5 represent clinicopathological predictors: HER2 status (purple is HER2-low, grey HER2-0, white unknown), density of TILs (percentage scored on H&E staining), presence of angioinvasion (brown indicating presence, grey absence), and clinical nodal status (pink indicating node positive, yellow node negative). Track 6 indicates the delta score of the expression of genes higher or lower expressed in the poor compared to the good responders (orange means a score >1 and green a score <1). LOO = Leave-one-out; HER2 = Human epidermal growth factor receptor 2; TIL = Tumor infiltrating lymphocytes; angio = angioinvasion; cN = clinical nodal status. (For interpretation of the references to colour in this figure legend, the reader is referred to the Web version of this article.)

To evaluate if a predictive gene expression signature was present in our RNA sequencing subset, a LOO procedure was used (Fig. 2A). After iterating all 78 samples and comparing the predicted status of each sample with the actual good/poor status, the model showed a 71% sensitivity to predict a poor responder (27 out of 38 poor responders) and an 83% specificity (33 out of 40 good responders). The positive/negative predictive values (PPV/NPV) were 79% and 75%, respectively. In total, 105 different genes were used throughout all iterations. The top genes that were most frequently used (in at least 50 test sets; 25 genes in total) are listed in supplementary file 2. Next, the expression levels of the top 25 genes of the prediction model were used to cluster all samples (Fig. 2B). Of the 25 genes, 13 genes were higher expressed in the poor responders. The clustering clearly differentiated between good and poor responders; all samples but one were divided perfectly by responder status over the two main sample clusters.

Lastly, it was evaluated whether these 25 most predictive genes from the LOO model could independently add to the above-described multivariate model including clinicopathologic characteristics. Reassuringly, three out of four identified multivariate clinicopathologic characteristics; clinical nodal status, HER2 status, and density of TILs remained independently significant within the smaller RNA sequencing subset (p < 0.001, p < 0.001 and p = 0.003, respectively). The 25 genes were combined into a single ‘delta’ score for each sample (see methods and Fig. 2B), and this delta score was added to the multivariate characteristics. This model with five features showed that clinical nodal status (node negative versus positive), HER2 status (HER2-0 versus HER2-low) and delta score remain independently significant (p < 0.001, p = 0.01, and p < 0.001, respectively), while angioinvasion and density of TILs are no longer independent predictive markers for the response to NAC (p = 0.471 and p = 0.953 respectively).

### 3.4 External validation of prediction model for response to NAC

**Fig. 3:**
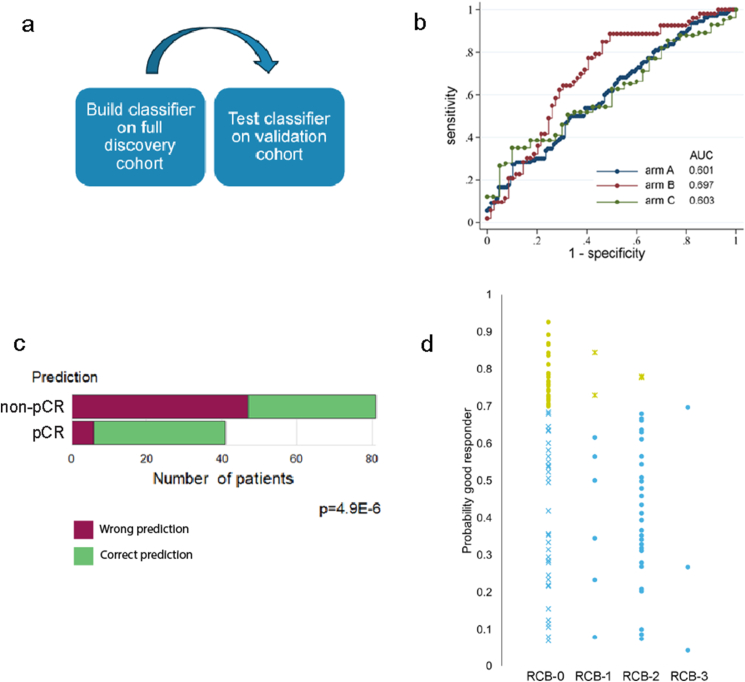
A 31-gene prediction model based on our dataset was validated in an independent study by Loibl et al. [21] A) Schematic overview of the study set-up for the validation. B) The ROC curves of the different treatment arms within the study. Treatment arm A received veliparib, carboplatin and paclitaxel, while patients in arm B received carboplatin and paclitaxel. The treatment of patients in arm C consisted of only paclitaxel. C) Prediction of response in the validation cohort, restricted to patients in arm B. D) Predicted probability of treatment response categorized by RCB group in patients from treatment arm B of the validation cohort. The cutoff score for prediction of a good responder was set at 0.7. All yellow points represent patients predicted to be good responders, whereas all blue data points represent patients predicted to have residual disease. Patients that were correctly predicted are presented with a dot, patients that were wrongly predicted are marked with an x. AUC = Area under the curve; pCR = pathological complete response. (For interpretation of the references to colour in this figure legend, the reader is referred to the Web version of this article.)

An independent dataset was used to validate the predictive power of a gene signature. With the use of our complete RNA sequencing subset as a discovery cohort, a new 31-gene signature was extracted and tested within the validation cohort of Loibl et al. (Fig. 3A and supplementary file 3) [21]. Within the validation cohort, patients were treated based on three treatment arms.

All patients within the validation cohort were analyzed and got a call for response (pCR versus non-pCR) following the 31-gene signature. Based on the Receiver Operating Characteristics (ROC) curve and given that arm B most closely resembled our cohort in terms of treatment, we selected this arm for further analysis (Fig. 3B). Patients in treatment arm B received paclitaxel (80 mg/m2 intravenously weekly for 12 doses) plus carboplatin (area under the curve 6 mg/mL per min, intravenously every 3 weeks, for four cycles) plus a veliparib placebo, twice a day. This regimen was followed by doxorubicin and cyclophosphamide every 2–3 weeks for four cycles. When looking at the predictive value of the 31-gene signature within this arm, using a predicted probability threshold of 0.7, 41 patients were predicted to be good responders, and 35 of these 41 were indeed good responders (85%; p = 4.9E-6; Fig. 3C). Since the definition for a poor responder in our own dataset differed from this external dataset (our poor responders were RCB2 and RCB3-like), we also analyzed the predictive value based on the amount of residual disease (RCB 0 = pCR, RCB 1-3 = limited, moderate and extensive amount of residual disease, respectively; Fig. 3D). In the RCB1 group, two out of 8 patients were wrongly predicted as having a pCR, compared to two out of 30 within the RCB2 group (RCB status was unknown for 16 patients). None of the three RCB3 cases were predicted as having a pCR. Looking at the patients that were predicted as having residual disease in arm B, only 47 out of 81 were correctly predicted (58%).

## 4 Discussion

Within this retrospective multicenter study, a gene expression prediction model was identified and validated, capable of predicting TNBC patients with a good response to NAC.

A supervised clustering analysis using only the expression of the top 25 genes from our dataset using the LOO design, revealed a clear stratification of samples into good and poor responders, which underscores the potential predictive power of these genes. In addition to this RNA-based prediction model, a combined RNA-histopathology model (including clinical nodal stage, angioinvasion, HER2 status and density of TILs) was investigated and showed that the clinical nodal stage, HER2 status and delta score, based on the expression of the genes in the prediction model, were independent predictors for a good response to NAC. However, since the LOO model already showed a near-perfect clustering of good and poor responders based on 25 genes, the model's performance could not be improved by including clinicopathologic variables like nodal stage or HER2 status (HER2-0 versus HER2-low).

To improve the clinical utility, our results were validated in an independent cohort [21]. Since clinicopathological variables from this external cohort were unavailable, only the gene expression profile could be validated. A 31-gene signature based on our complete dataset demonstrated promising predictive power for having a pCR in the validation set (35 out of 41 patients were correctly predicted; 85%), using the patient population with the most similar treatment regimen. Notably, the proportion of patients with a limited amount of residual disease (RCB1) was relatively high among those 6 cases that were wrongly predicted as a pCR and none of the RCB3 cases were predicted as having a pCR. This suggests that tumors from patients with a pCR and RCB1 exhibit more overlapping gene expression profiles compared to tumors from patients with more extensive residual disease. The prediction of having residual disease on the other hand was limited upon external validation. This discrepancy is likely attributable to differences in patient characteristics across the cohorts. In our cohort, good responders were defined as having a pCR without a recurrence during follow-up. This definition closely resembles that used in the validation cohort, which likely explains the good predictive performance of the model in this group of patients. In contrast, the definition of a poor responder in our dataset differed substantially from the one used in the validation set. In our cohort, poor responders were RCB3-like, while the validation cohort included all patients with a non-pCR. This likely underlies the poor predictive accuracy in the whole group of non-pCR patients from the validation cohort. Interestingly, despite the limitation of small numbers of RCB-3 cases, the predictive performance of the 31-gene signature seemed encouraging in this group of patients, underscoring its potential for identifying the most chemo-resistant tumors in a comparable patient population.

The prediction model based on our own sample set (25 genes) and the prediction model based on the validation dataset (31 genes) showed a high number of overlaps in genes. Only two genes that were in our original 25-gene model were not included in the validation prediction model (*NOCA1* and *CCDC9B*). This overlap is reassuring that the genes are relevant predictors for the response to NAC. Collectively, genes in the prediction model define a stress-adaptive cellular state characterized by cytokine-driven signaling (e.g. *STAT3*) [22], regulated immune engagement (e.g. *IFI16*) [23], and metabolic flexibility (e.g. *SLC43A2*, *FAAH*) [24,25]. The presence of DNA repair and RNA regulatory factors (e.g. *RECQL*, *MATR3*) [26,27] suggest maintained cellular fitness rather than irreversible resistance. On the other hand, membrane trafficking and junctional components, like *CLDN20*, indicate the promotion of aggressive phenotypes through extracellular cues [28].

A recent study by Martin et al. reported one of the first clinical genomic tests tailored specifically for early-stage TNBC, the TNBC-DX assay [29]. Although there was no overlap between the gene sets used in our study and the TNBC-DX assay, both incorporated markers related to immune response and cellular proliferation. A novelty of our study was that we compared the ‘extreme’ groups of patients with either a very good or very poor clinical outcome.

Following the promising results of the KEYNOTE-522 study, clinical practice is moving towards the combination of NAC and immunotherapy for stage II/III TNBC patients [6,30]. However, despite its benefits in a subset of patients, pembrolizumab carries a considerable risk of immune-related toxicity. Our model enables the identification of patients most likely to achieve favorable outcomes based on NAC alone. In such cases, withholding pembrolizumab could reduce overtreatment and healthcare burden without compromising efficacy.

In addition, high abundance of TILs is associated with the response rate to NAC, but also with improved prognosis, which may enable further de-escalation by omitting chemotherapy in a subset of TIL-high TNBC patients [1,4,31]. On the other hand, poor responders more often had a HER2-low tumor, so these patients could benefit from antibody-drug conjugates, like trastuzumab deruxtecan [32]. This observation is consistent with previous reports linking HER2-0 tumors to a more immunogenic tumor microenvironment and higher proliferative activity, characteristics that may underlie their increased chemosensitivity [33,34].

The inclusion of patients from multiple centers throughout the Netherlands resulted in a unique cohort of patients characterized by either a very good or very poor responses to NAC. However, our strict inclusion criteria hampered finding an external dataset resembling our own, which likely resulted in the observed poor performance in patients with residual disease. In addition, the chemotherapy regimens were heterogenous, and we did not perform central review for the HER2 marker, which could have affected our results since HER2-low is known to have high interobserver disagreement [35,36].

## 5 Conclusion

In summary, this study indicates that a multigene prediction model may help identify TNBC patients likely to achieve a pCR after NAC. For those with a predicted pCR based on NAC alone, omission of immunotherapy could be explored as a strategy to reduce immune-related toxicity. Nonetheless, while these findings are encouraging, further optimization and prospective validation are essential before progressing towards clinical implementation.

## CRediT authorship contribution statement

**Nadine S. van den Ende:** Writing – original draft, Visualization, Validation, Methodology, Investigation, Formal analysis, Data curation, Conceptualization. **Marcel Smid:** Writing – review & editing, Visualization, Validation, Methodology, Formal analysis, Data curation. **John W.M. Martens:** Writing – review & editing, Conceptualization. **Reno Debets:** Writing – review & editing, Conceptualization. **Agnes Jager:** Writing – review & editing, Conceptualization. **Carolien H.M. van Deurzen:** Writing – review & editing, Supervision, Funding acquisition, Conceptualization.

## Ethics approval

Coded, leftover patient material was used in accordance with the Code of Conduct of the Federation of Medical Scientific Societies in the Netherlands [12]. According to these national guidelines, this work was not subject to the Medical Research Involving Human Subjects Act (WMO; METC 02.593).

## Declaration of competing interest

C.H.M*.* van Deurzen received research funding from AstraZeneca and Roche, but this was unrelated to this project. R. Debets has received research support from MSD and Bayer, personal fees from Bluebird Bio, Genticel, other support from Pan Cancer T outside the submitted work (all paid to the Erasmus MC Cancer Institute), as well as European patent application no's 21152822.9, 24192717.7 and 25163944.9 (pending to Erasmus MC). J.W.M. Martens received research funding from Menarini, Tzu genomics, MSD and Pfizer and a consultancy fee from Novartis; these were all unrelated to this project. All the other authors declare no potential competing interests.

## Data Availability

The data generated in this study are available within the article and its supplementary data files. Raw data is available upon request from the corresponding author.
