# Supplementary material for: A multigene model for response stratification to neoadjuvant chemotherapy in triple negative breast cancer
Source: Breast. 2026 Mar 17;87:104764. doi: 10.1016/j.breast.2026.104764 (PMC13019104; doi:10.1016/j.breast.2026.104764)
Supplement: Multimedia component 1 [file mmc1.docx]

| Treatment | Good responders (n=87, 43%) | Poor responders (n=117, 57%) |
| --- | --- | --- |
| Cyclofosphamide |  |  |
| Yes | 70 (80%) | 56 (48%) |
| No | 12 (14%) | 54 (46%) |
| Unknown | 5 (6%) | 7 (6%) |
| Immunotherapy |  |  |
| Yes | 0 (0%) | 3 (3%) |
| No | 82 (94%) | 107 (91%) |
| Unknown | 5 (6%) | 7 (6%) |
| Anthracycline |  |  |
| Yes | 82 (94%) | 99 (84%) |
| No | 0 (0%) | 11 (9%) |
| Unknown | 5 (6%) | 7 (6%) |
| Taxane |  |  |
| Yes | 82 (94%) | 95 (81%) |
| No | 0 (0%) | 15 (13%) |
| Unknown | 5 (6%) | 7 (6%) |
| Platinum agents |  |  |
| Yes | 47 (54%) | 38 (32%) |
| No | 35 (40%) | 72 (62%) |
| Unknown | 5 (6%) | 7 (6%) |
